# Structural Violence and Clinical Medicine

**DOI:** 10.1371/journal.pmed.0030449

**Published:** 2006-10-24

**Authors:** Paul E Farmer, Bruce Nizeye, Sara Stulac, Salmaan Keshavjee

## Abstract

Structural violence refers to the social structures that put people in harm's way. Farmer and colleagues describe the impact of social violence upon people living with HIV in the US and Rwanda.

Because of contact with patients, physicians readily appreciate that large-scale social forces—racism, gender inequality, poverty, political violence and war, and sometimes the very policies that address them—often determine who falls ill and who has access to care. For practitioners of public health, the social determinants of disease are even harder to disregard.

Unfortunately, this awareness is seldom translated into formal frameworks that link social analysis to everyday clinical practice. One reason for this gap is that the holy grail of modern medicine remains the search for the molecular basis of disease. While the practical yield of such circumscribed inquiry has been enormous, exclusive focus on molecular-level phenomena has contributed to the increasing “desocialization” of scientific inquiry: a tendency to ask only biological questions about what are in fact *biosocial* phenomena [1].

Biosocial understandings of medical phenomena are urgently needed. All those involved in public health sense this, especially when they serve populations living in poverty. Social analysis, however rudimentary, occurs at the bedside, in the clinic, in field sites, and in the margins of the biomedical literature. It is to be found, for example, in any significant survey of adherence to therapy for chronic diseases [2,3] and in studies of what were once termed “social diseases” such as venereal disease and tuberculosis (TB) [4–8]. The emerging phenomenon of acquired resistance to antibiotics—including antibacterial, antiviral, and antiparasitic agents—is perforce a biosocial process, one which began less than a century ago as novel treatments were introduced [9]. Social analysis is heard in discussions about illnesses for which significant environmental components are believed to exist, such as asthma and lead poisoning [10–15]. Can we speak of the “natural history” of any of these diseases without addressing social forces, including racism, pollution, poor housing, and poverty, that shape their course in both individuals and populations? Does our clinical practice acknowledge what we already know—namely, that social and environmental forces will limit the effectiveness of our treatments? Asking these questions needs to be the beginning of a conversation within medicine and public health, rather than the end of one.

**Box 1. What Is Structural Violence?**

*Structural violence*, a term coined by Johan Galtung and by liberation theologians during the 1960s, describes social structures—economic, political, legal, religious, and cultural—that stop individuals, groups, and societies from reaching their full potential [57]. In its general usage, the word *violence* often conveys a physical image; however, according to Galtung, it is the “avoidable impairment of fundamental human needs or…the impairment of human life, which lowers the actual degree to which someone is able to meet their needs below that which would otherwise be possible” [58]. Structural violence is often embedded in longstanding “ubiquitous social structures, normalized by stable institutions and regular experience” [59]. Because they seem so ordinary in our ways of understanding the world, they appear almost invisible. Disparate access to resources, political power, education, health care, and legal standing are just a few examples. The idea of *structural violence* is linked very closely to *social injustice* and the social machinery of oppression [16].

## Defining Structural Violence

The term “structural violence” is one way of describing social arrangements that put individuals and populations in harm's way (see Box 1) [16]. The arrangements are *structural* because they are embedded in the political and economic organization of our social world; they are *violent* because they cause injury to people (typically, not those responsible for perpetuating such inequalities). With few exceptions, clinicians are not trained to understand such social forces, nor are we trained to alter them. Yet it has long been clear that many medical and public health interventions will fail if we are unable to understand the social determinants of disease [17,18].

The good news is that such biosocial understandings are far more “actionable” than is widely recognized. There is already a vast and growing array of diagnostic and therapeutic tools born of scientific research; it is possible to use these tools in a manner informed by an understanding of structural violence and its impact on disease distribution and on every step of the process leading from diagnosis to effective care. This means working at multiple levels, from “distal” interventions—performed late in the process, when patients are already sick—to “proximal” interventions—trying to prevent illness through efforts such as vaccination or improved water and housing quality.

As with many other concepts, structural violence has its limitations [19]. Nevertheless, we seek to apply the concept to what remain the primary tasks of clinical medicine: preventing premature death and disability and improving the lives of those we care for. Using the concept of structural violence, we intend to begin, or revive, discussions about social forces beyond the control of our patients.

These forces are not beyond the reach, however, of practitioners of medicine and public health. In this article, we describe examples of the impact of structural violence upon people living with HIV in the United States and in Rwanda. In both cases, we show that it is possible to address structural violence through structural interventions. We then draw general lessons from these examples for health professionals and policy makers worldwide.

## Delivering AIDS Care Equitably in the United States

The distribution and outcome of chronic infectious diseases, such as HIV/AIDS, are so tightly linked to social arrangements that it is difficult for clinicians treating these diseases to ignore social factors. Although AIDS is often considered a “social disease,” clinicians may have radically different understandings of what makes AIDS “social.” Many doctors have focused on the “behaviors” or “lifestyles” that place some at risk for HIV infection [20–23]. Yet risk has never been determined solely by individual behavior: susceptibility to infection and poor outcomes is aggravated by social factors such as poverty, gender inequality, and racism [24–26]. Unsurprisingly, in less than a decade AIDS became a disease that disproportionately affected America's poor, many of whom engaged in “risk behaviors” at a far lower rate than others who were not at heightened risk of infection with sexually transmitted diseases [27–29].

### Factors affecting disease course.

HIV attacks the immune system in only one way, but its course and outcome are shaped by social forces having little to do with the universal pathophysiology of the disease. From the outset of acute HIV infection to the endgame of recurrent opportunistic infections, disease course is determined by, to cite but a few obvious factors: (1) whether or not postexposure prophylaxis is available; (2) whether or not the steady decline in immune function is hastened by concurrent illness or malnutrition; (3) whether or not multiple HIV infections occur; (4) whether or not TB is prevalent in the surrounding environment; (5) whether or not prophylaxis for opportunistic infections is reliably available [30]; and (6) whether or not antiretroviral therapy (ART) is offered to all those needing it.

Throughout the usually decade-long process of HIV progression, detrimental social structures and constructs—structural violence—have a profound influence on effective diagnosis, staging, and treatment of the disease and its associated pathologies. Each of these determinants of disease course and outcome is itself shaped by the very social forces that determine variable risk of infection.

Although the variability of outcomes has been especially obvious in the era of effective therapy, it was so even before ART became widely available. In Baltimore in the early 1990s, Moore et al. showed that race was associated with the timely receipt of therapeutics: among patients infected with HIV, blacks were significantly less likely than whites to have received ART or Pneumocystis pneumonia prophylaxis when they were first referred to an HIV clinic, regardless of disease stage at the time of presentation [31]. The timeline from HIV infection to death was further shortened in situations where TB was the leading opportunistic infection, as it is in much of the poor world [32]. These fundamentally biosocial events call into question a “natural history” of HIV infection and AIDS.

### Addressing disparities in HIV care.

In an attempt to address these ethnic disparities in care, researchers and clinicians in Baltimore reported how racism and poverty—forms of structural violence, though they did not use these specific terms—were embodied [33,34] as excess mortality among African Americans without insurance. After documenting these disparities, these clinicians and researchers asked: what would happen if race and insurance status no longer determined who had access to the standard of care?

Their subsequent interventions were decidedly proximal: in addition to removing some of the obvious economic barriers at the point of care, the clinicians and researchers considered paying for transportation costs and other incentives as well as addressing comorbid conditions ranging from drug addiction to mental illness. They also implemented improvements in community-based care, conceived to make AIDS care more convenient and socially acceptable for patients. The goal was to make sure that nothing within the medical system or the surrounding community prevented poor and otherwise marginalized patients from receiving the standard of care.

The results registered just a few years later were dramatic: racial, gender, injection-drug use, and socioeconomic disparities in outcomes largely disappeared within the study population [35]. In other words, these program improvements may not have dealt with the lack of national health insurance, and still less with the persistent problems of racism and urban poverty, but they did lessen the embodiment of social inequalities as premature death from AIDS. Similar work elsewhere has shown the ability of providers to lessen the impact of social inequalities on AIDS outcomes among the homeless, the addicted, the mentally ill, and prisoners [36–38].

The program in Baltimore was improved in part by linking an understanding of social context to clinical services. The importance of such programs is underscored by the emergence of multidrug-resistant HIV in the United States [39]. Microbial acquisition of resistance to antibiotics, including antiretrovirals, is necessarily a biosocial phenomenon. Most microbes mutate when challenged with antibiotics; the rate of mutation may be hastened by imprudent use of antibiotics or by inadequate or interrupted therapy [40,41]. Although structural violence lessens both access and adherence to effective therapy, it is a rarely discussed contributor to epidemics of multidrug-resistant HIV. In reality, it is impossible to understand the dynamics of drug-resistant disease without understanding how structural violence is embodied at the community, individual, and microbial levels [9,42]. The lessons from Baltimore show us that by viewing access to care and adherence to treatment as structural issues requiring programmatic solutions, we can alter the very biology of HIV and the “natural history” of AIDS.

## Preventing Pediatric AIDS in Rwanda: Lessons from Rural Haiti

The impact of structural violence is even more obvious in the world's poorest countries and has profound implications for those seeking to provide clinical services there. Over the past year, working with the nonprofit organization Partners In Health (PIH), we have sought to address AIDS and TB in Africa, the world's poorest and most heavily burdened continent. Specifically, we have transplanted and adapted the “PIH model” of care, which was designed in rural Haiti to prevent the embodiment of poverty and social inequalities as excess mortality due to AIDS, TB, malaria, and other diseases of poverty [43,44].

### The PIH model.

In some senses, the model is simple: clinical and community barriers to care are removed as diagnosis and treatment are declared a public good and made available free of charge to patients living in poverty. Furthermore, AIDS care is delivered not only in the conventional way at the clinic, but also within the villages in which our patients work and live.

Each patient chooses an *accompagnateur*, usually a neighbor, trained to deliver drugs and other supportive care in the patient's home. Using this model, we currently provide daily supervised ART to more than 2,200 patients in rural Haiti. This model, with conventional clinic-based (distal) services complemented by home-based (more proximal) care, is deemed by some to be the world's most effective way of removing structural barriers to quality care for AIDS and other chronic diseases. It is also a way of creating jobs in rural regions in great need of them. We have used a similar model in urban Peru [45,46], and in Boston, Massachusetts [37].

### The challenge of HIV in Rwanda.

Rwanda presents unique challenges, but many barriers to care are quite similar to those seen in Haiti and other settings where social upheaval, poverty, and gender inequality decrease the effectiveness of distal services and of prevention efforts. Like Haiti, Rwanda is a densely populated, predominantly agrarian society. Although both countries have endured large-scale political violence, that which registered a decade ago in Rwanda due to war and genocide was unprecedented in scale. In the two rural districts of Rwanda in which the PIH model was introduced in May 2005, an estimated 60 percent of inhabitants are refugees, returning exiles, or recent settlers; not a single physician was present to serve 350,000 people.

AIDS has recently worsened this situation and is a leading cause of young adult death. In spite of the availability of significant resources to treat complications of HIV infection in Africa, almost all patients enrolled on ART live in cities or towns. Indeed, some have noted that rapid treatment scale-up is likely to occur largely in urban settings, where infrastructure, though poor, is better than in rural regions [47]. The challenge, however, is to reach rural Africa, where fewer than five percent of those who need ART receive it. Rural treatment scale-up is far from impossible: less than a year after our program began in 2005, more than 1,500 rural Rwandans with AIDS were already enrolled in care using the PIH model.

To deepen our discussion of interventions designed to counter structural violence, consider the prevention of mother-to-child transmission (MTCT) of HIV in rural Rwanda. Where clean water is unavailable and HIV prevalence is high, the policy of universal breast-feeding—driven by the desire to reduce diarrhea-related mortality—leads to increased transmission of the virus to infants, even when ART is offered. We knew from our experience in Haiti that we could reduce rates of MTCT from as high as 25 to 40 percent to as low as two percent. We knew that such a dramatic reduction could be made possible by: (1) providing combination ART to the mother during pregnancy; (2) enabling formula-feeding and close follow-up of infants; and (3) launching potable water projects within the catchment area—in even the most difficult regions, where electricity is scarce, food insecurity widespread, and health and sanitation infrastructure rudimentary at best [48].

Although our pilot project in Rwanda is only a year old, its feasibility is almost certain. In the first six months of operation, we screened for HIV infection more than 31,000 persons in the two districts in which we work. Without exception, pregnant women found to be infected with HIV expressed interest in ART to prevent MTCT, and all requested assistance not only with procuring infant formula, but also with the means to boil water and to store the formula safely (Figure 1).

**Figure 1:**
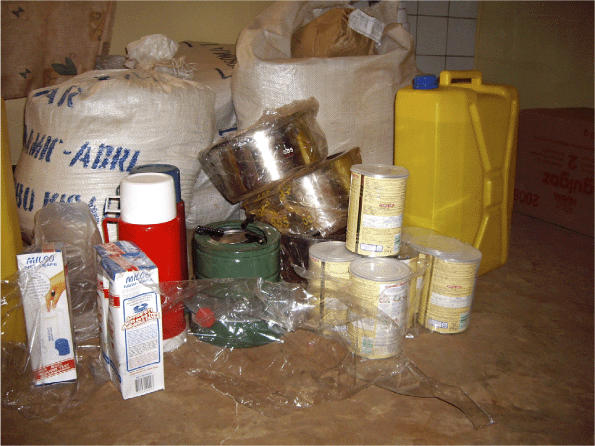
Components of the Package for Prevention of Mother-to-Child Transmission of HIV Provided by Partners In Health/Inshuti Mu Buzima in Rwanda Photo: Ophelia Dahl, Partners In Health

Medical professionals are not trained to make structural interventions.

Our distal intervention is to provide ART to all women in the catchment area with the help of *accompagnateurs*. More proximal interventions include the distribution of kerosene stoves, kerosene, bottles, and infant formula; we also provide food aid and housing assistance when possible. Already, we are seeing a lowering of HIV infection rates amongst newborns, and we believe that, as the program becomes well established and services become available earlier during the course of pregnancy, rates of MTCT will continue to decline.

Unsurprisingly, opposition to the PIH model did not come from rural Rwandan women living with HIV. Rather, we faced the most resistance to this approach from local and global health policy makers who continued to promote universal breast-feeding, a policy which made eminent sense prior to the advent of HIV. Instead of trying to overcome programmatic barriers, the experts argued that formula-feeding was simply not feasible in rural Rwanda and that HIV-related stigma would prevent women from enrolling in such projects.

The examples of Rwanda and Haiti have shown us that, to date, there is little reason to believe that thoughtful structural interventions will fail to improve HIV prevention and treatment outcomes. Any failure is more likely to be due to programmatic shortfalls than to stigma or to non-compliance on the part of the patients enrolled in the program. Structural interventions of the sort described here remove the onus of adherence from vulnerable patients and place it squarely on the health system and on providers.

## Incorporating Structural Interventions in Medicine and Public Health

If structural violence is often a major determinant of the distribution and outcome of disease, why is it or a similar concept not in wider circulation in medicine and public health, especially now that our interventions can radically alter clinical outcomes? One reason is that medical professionals are not trained to make structural interventions. Physicians can rightly note that structural interventions are “not our job.” Yet, since structural interventions might arguably have a greater impact on disease control than do conventional clinical interventions, we would do well to pay heed to them.

Acknowledging and addressing structural impediments, however, should never be the sole focus of our work. For decades, those who study the determinants of disease have known that social or structural forces account for most epidemic disease. But truisms such as “poverty is the root cause of tuberculosis” have not led us very far. While we do not yet have a curative prescription for poverty, we do know how to cure TB. Those who argue that focusing solely on economic development will in time wipe out tuberculosis may be correct, but en route toward this utopia the body count will remain high if care is not taken to diagnose and treat the sick. The same holds true for other diseases of poverty. Clean water and sanitation will prevent cases of typhoid fever, but those who fall ill need antibiotics; clean water comes too late for them.

The debate about whether to focus on proximal versus distal interventions, or similar debates about how best to use scarce resources, is as old as medicine itself. But there is little compelling evidence that we must make such either/or choices: distal and proximal interventions are complementary, not competing. International public health is rife with false debates along precisely these lines, and the list of impossible choices facing those who work among the destitute sick seems endless. In reality, there is no good way to tackle the health crisis in Africa when the scant resources previously available are so bitterly contested; thus is structural violence perpetuated at a time in which science and medicine continue to yield truly miraculous tools. Without an equity plan to bring these tools to bear on the health problems of the destitute, these debates will continue to waste precious time [49].

### The lessons of Baltimore, Haiti, and Rwanda.

What are the lessons that can be drawn from the examples of successful structural interventions in the diverse settings of Baltimore, rural Haiti, and rural Rwanda? First, we have seen that it is possible to decrease the extent to which social inequalities become embodied as health disparities. While some interventions are straightforward, we also have to recognize that there is an enormous flaw in the dominant model of medical care: as long as medical services are sold as commodities, they will remain available only to those who can purchase them. National health insurance and other social safety nets, including those that guarantee primary education, food security, and clean water, are important because they promise rights, rather than commodities, to citizens. The lack of these social and economic rights is fundamental to the perpetuation of structural violence [50].

Second, we have learned that proximal interventions, seemingly quite remote from the practice of clinical medicine, can also lessen premature morbidity and mortality. To put this in sociological terms, interventions that increase the agency of the poor will lessen their risk of HIV. Similarly, it is not possible to have an honest discussion of alcoholism among Native Americans [51], or crack cocaine addiction among African Americans [52], without discussing the history of genocide and slavery in North America. Again, such commentary is often seen as extraneous in medical and public health circles, where discussions of substance abuse are curiously desocialized, viewed as personal and psychological problems rather than societal ones. Here, too, structural violence is perpetuated through analytic omission.

Third, we have seen that structural interventions can have an enormous impact on outcomes, even in the face of cost-effectiveness analyses and the flawed policies of international bodies. Taking the components of the distal interventions already underway in Rwanda—infant formula, clean water, fuel, and so forth—it is possible to go further and describe more proximal interventions to improve access to each component of the project. These would include, of course, legislation to promote generic medications, improved distribution networks for ART and infant formula, clean-water campaigns, and the development of alternative fuels. More proximally still, they would include enhancing agricultural production; creating new jobs outside of the agricultural sector; addressing gender inequality through legislation about land tenure and political representation [53]; and promoting adult literacy.

These are not the tasks for which clinicians were trained, but they are central to the struggle to reduce premature suffering and death. The importance of structural interventions for the future of health care means that practitioners of medicine and public health must make common cause with others who are trained to intervene more proximally. Sometimes public health crises, such as the AIDS pandemic in Africa, can lead to bold and specific interventions, such as the campaign to provide AIDS prevention and care as a public good [54]. When linked to more structural interventions, such ostensibly specific campaigns can help to trigger a “virtuous social cycle” that promises to shift the burden of pathology away from children and young adults—a major victory in the struggle to lessen structural violence.

## Conclusions

Pioneers of modern public health during the nineteenth century, such as Rudolph Virchow, understood that epidemic disease and dismal life expectancies were tightly linked to social conditions [55,56]. Such leaders might not have employed the term “structural violence,” but they were well aware of its toll and argued compellingly for proximal interventions: education, basic sanitation, land reform, sovereignty, and an end to political oppression. These interventions are no less needed now that we have better distal tools, including vaccines, diagnostics, and a large armamentarium of effective therapeutics.

It does not matter what we call it: structural violence remains a high-ranking cause of premature death and disability. We can begin to address this by “resocializing” our understanding of disease distribution and outcome. Even new diseases such as AIDS have quickly become diseases of the poor, and the development of effective therapies may have a perverse effect if we are unable to use them where they are needed most. By insisting that our services be delivered equitably, even physicians who work on the distal interventions characteristic of clinical medicine have much to contribute to reducing the toll of structural violence. The poor are the natural constituents of public health, and physicians, as Virchow argued, are the natural attorneys of the poor. In this struggle, equity in healthcare *is* our responsibility. Only when we link our efforts to those of others committed to initiating virtuous social cycles can we expect a future in which medicine attains its noblest goals.

## Abbreviations

ART: antiretroviral therapy
MTCT: mother-to-child transmission
PIH: Partners In Health
TB: tuberculosis
